# Costs associated with policies regarding alcohol use during pregnancy: Results from 1972-2015 Vital Statistics

**DOI:** 10.1371/journal.pone.0215670

**Published:** 2019-05-08

**Authors:** Meenakshi S. Subbaraman, Sarah C. M. Roberts

**Affiliations:** 1 Alcohol Research Group, Public Health Institute, Emeryville, CA, United States of America; 2 Advancing New Standards in Reproductive Health (ANSIRH), Bixby Center for Global Reproductive Health, Department of Obstetrics, Gynecology & Reproductive Sciences, University of California San Francisco, Oakland, CA, United States of America; Agha Khan University, UNITED REPUBLIC OF TANZANIA

## Abstract

**Background and objective:**

As of 2016, 43 US states have policies regarding alcohol use during pregnancy. A recent study found that out of eight state-level alcohol/pregnancy policies, six are significantly associated with poorer birth outcomes, and two are not associated with any outcomes. Here we estimate the excess numbers of low birthweight (LBW) and preterm births (PTB) related to these policies and their associated additional costs in the first year of life.

**Methods:**

Cost study using birth certificate data for 155,446,714 singleton live births in the United States between 1972–2015. Exposures were state- and month/year-specific indicators of having each of eight alcohol/pregnancy policies in place. Outcomes were excess numbers of LBW and PTB and associated costs in the first year of life. Fixed effects regressions with state-specific time trends were used for statistical analyses in 2018.

**Results:**

In 2015, policies mandating warning signs were associated with an excess of 7,375 LBW; policies defining alcohol use during pregnancy as child abuse/neglect were associated with an excess of 12,372 PTB; these excess adverse outcomes are associated with additional costs of $151,928,002 and $582,698,853 in the first year of life, respectively.

**Conclusions:**

Multiple state-level alcohol pregnancy policies lead to increased prevalence of LBW and PTB, which cost hundreds of millions of dollars annually. Policymakers should consider adverse public health impacts of alcohol/pregnancy policies before expanding extant policies to new substances or adopting existing policies in new states.

## Introduction

As of 2016, 43 US states have policies regarding alcohol use during pregnancy [1]. These include mandatory warning signs (MWS), giving pregnant women priority for substance abuse treatment (PTPREG), giving pregnant women and women with children priority for substance abuse treatment (PTPREGWC), requiring reporting for either child welfare purposes (RRCPS) or data collection and treatment purposes (RRDTx), limiting criminal prosecution (LCP), allowing civil commitment (CC), and defining drinking during pregnancy as child abuse/neglect (CACN). Most of these, with the exception of MWS, apply to both alcohol and drug use during pregnancy [1].

Many of these policies have been in effect for decades, some for more than forty years [2]. Policy activity on these topics continues in both state legislatures and in the courts. For example, in 2019, the Michigan legislature is considering adopting a MWS policy for alcohol and the Tennessee legislature is considering re-adopting a law criminalizing drug use during pregnancy [3, 4]. Other states are expanding extant policies to cover new substances, e.g., coinciding with state-level cannabis legalization, a few states have expanded MWS policies to include cannabis [1]. State policies are being challenged in court as well; in December 2018, a legal challenge to the Pennsylvania CACN law as it related to opioid use during pregnancy resulted in the Pennsylvania Supreme Court ruling that behavior (e.g., substance use) while pregnant does not constitute child abuse under state law [5].

While policy activity on this topic continues, a recent study suggests that state legislators typically do not consider research evidence in their policy-making related to alcohol and drug use during pregnancy [6]. Among many reasons for the lack of evidence in public health policy-making in general, an especially important issue related to policies regarding substance use during pregnancy is that, until recently, there has been little research examining the impact of these policies on either pregnant women or their infants. Furthermore, most of the previous research about state-level policy impacts has considered each policy in isolation. For example, a few qualitative studies have found that fear of being reported to Child Protective Services (CPS) is a reason women who use alcohol and/or drugs avoid prenatal care [7, 8]. A previous study on MWS found that MWS may be associated with reductions in very low birthweight [9], although that study did not control for other policies in effect at the same time and did not account for the month and year the policies went into effect. While not directly related to MWS, other research has found that the fear of having already irreversibly harmed her baby from substance use is a reason women avoid prenatal care and/or do not reduce or stop their use later in pregnancy [7, 10].

Only one study has comprehensively assessed impacts of these policies, finding that most alcohol/pregnancy policies are not associated with alcohol use during pregnancy, and that those that are associated in different directions [11]. This study also found that most alcohol/pregnancy policies lead to increases in adverse birth outcomes, perhaps because some also lead to decreases in prenatal care utilization [12]. Regarding birth outcomes, out of eight policies in effect in 2013, six were significantly associated with poorer birth outcomes and/or less prenatal care, and two were not associated with any outcomes [12]. The most consistent effects were found for pregnant women living in states with MWS and CACN policies, which both led to higher odds of low birthweight (LBW), preterm birth (PTB), no or late prenatal care, and lower odds of normal APGAR scores. For example, living in a state with MWS was related to 7% higher odds of LBW (P < 0.001), 4% higher odds of PTB (P < 0.004), and 18% lower odds of any prenatal care (P < 0.001) compared to living in a state without MWS. Living in a state with CACN was related to 6% higher odds of LBW (P < 0.003), 9% higher odds of PTB (P < 0.001), and 13% lower odds of any prenatal care (P < 0.046) compared to living in a state without CACN [12]. Together the results suggest that alcohol/pregnancy policies may scare women who drink during pregnancy such that they avoid prenatal care utilization, which may contribute to worse birth outcomes, an explanation consistent with previous qualitative research [7, 8]. Although the magnitudes of the point estimates in this study were small, with statistically significant odds ratios related to LBW and PTB ranging from 1.05–1.11, they are likely to still be meaningful in a large population (N = 148,048,208) [12].

Still, the question remains as to what these findings mean from a public health perspective. While harms related to substance use during pregnancy come from the use itself [13, 14], it also appears that harms also come from policies adopted in response to alcohol and drug use during pregnancy. To assess whether the harms from the policies are significant from a public health perspective and not just statistically significant, it is important to translate odds ratios to units meaningful to policymakers–specifically to numbers of babies affected and to costs. Thus, we extend these earlier findings here by adding additional years of data and estimating the excess numbers of LBW and PTB under each policy for 2015 and their associated additional costs in the first year of life.

## Methods

### Data sources

Vital Statistics data from 1972–2004 are publicly available for download. Limited use and restricted Vital Statistics data from 2005–2015 are available from the CDC’s National Center for Health Statistics. We obtained limited use and restricted use datasets from the CDC for the years 2005–2015. Those datasets no longer include information such as exact dates and are thus anonymized. This research was considered Not Human Subjects Research by the University of California, San Francisco and Public Health Institute institutional review boards.

Birth certificate data were obtained from the Vital Statistics System for 155,446,714 singleton live births between 1972–2015; analyses were restricted to singleton births because multiples are known to be at higher risk of LBW and PTB [15], and to follow methodological criteria from previously published results [12]. These data were combined with alcohol/pregnancy policy data obtained from the National Institute on Alcohol Abuse and Alcoholism’s Alcohol Policy Information System (http://alcoholpolicy.niaaa.nih.gov/) and original legal research [2], along with other state-level control variables. Through 2015, eight policies targeting alcohol use among pregnant women were in effect in at least one US state: MWS, PTPREG, PTPREGWC, RRCPS, RRDTx, LCP, CC, and CACN.

Hospital costs estimates for additional costs due to LBW or PTB in the first year of life come from two primary sources. First, the Healthcare Cost and Utilization Project used hospital discharge data to show that costs for LBW/PTB admissions totaled $5.8 billion in one year, with costs for LBW and very LBW (<1500 g) births averaging $20,600 and $52,300 respectively [16]. Second, a study of private health insurance claims data calculated first-year expenditures for PTB infants in 2013 [17]. Conservative estimates showed that care in the first year of life for a PTB infant costs $47,100 per infant, while an alternative algorithm estimated that costs could be up to $78,000 per preterm infant [17]. We used the most conservative lower bounds of the published cost estimates in all analyses.

### Statistical analyses

Two logistic regressions analogous to those published [12] were fit with policy indicators as the exposure and odds of LBW and odds of PTB as outcomes. Each policy indicator was coded as 0 if it was not in effect for the mother’s state of residence during the month/year of conception and 1 if it was in effect during the month/year of conception; this method of linking the policy indicators to the month and year of conception improves the accuracy of exposure timing [18]. Both regressions adjusted for all policies simultaneously, as well as individual- and state-level covariates, fixed effects for state and year, and state-specific time trends; previously published analyses found no differences when controlling for all policies together simultaneously vs. each policy individually [12]. Individual-level covariates were maternal age, race, marital status, education, nativity, parity, and version of birth certificate. State-level covariates were state- and year- specific poverty, unemployment, per capita cigarette consumption, and per capita total ethanol consumption, as well as indicators for whether government control of wine sales and government control of spirit sales were in effect for that state in that year.

Predicted margins for each significant (*P*<0.05) policy were calculated and used to compute the excess proportion of low birthweight and preterm births under each significant policy. These proportions and their 95% confidence intervals were then applied to the number of births for 2015 in all U.S. states to estimate the number of excess LBW and PTBs in 2015. Finally, using marginal effects and the most conservative lower bounds of published cost estimates obtained from [16, 17], costs associated with excess LBW and PTB under each policy were estimated.

## Results

Multivariable logistic regressions showed that four policies were significantly (*P* < 0.05) related to increases in LBW and PTB: mandatory warning signs, giving pregnant women priority for substance abuse treatment, limits on criminal prosecution, and defining substance use during pregnancy as child abuse/neglect. Specifically, 1) mandatory warning signs led to a 0.3% (95% CI: 0.2%, 0.5%) increase in LBW and a 0.3% (95% CI: 0.1%, 0.6%) increase in PTB; 2) priority treatment for pregnant women led to a 0.5% (95% CI: 0.3%, 0.7%) increase in LBW and a 0.6% (95% CI: 0.2%, 1.1%) increase in PTB; 3) limits on criminal prosecution led to a 0.5% (95% CI: 0.1%, 0.8%) increase in LBW and a 0.9% (95% CI: 0.4%, 1.5%) increase in PTB; and finally, 4) policies defining substance use during pregnancy as child abuse/neglect led to 0.3% (95% CI: 0.1%, 0.6%) increase in LBW and a 0.7% (95% CI: 0.4%, 1.1%) increase in PTB. The four remaining policies, i.e., civil commitment, reporting requirements for either CPS or data/treatment purposes, and prioritizing treatment for pregnant women and women with children were no significantly related to either LBW or PTB.

**Fig 1:**
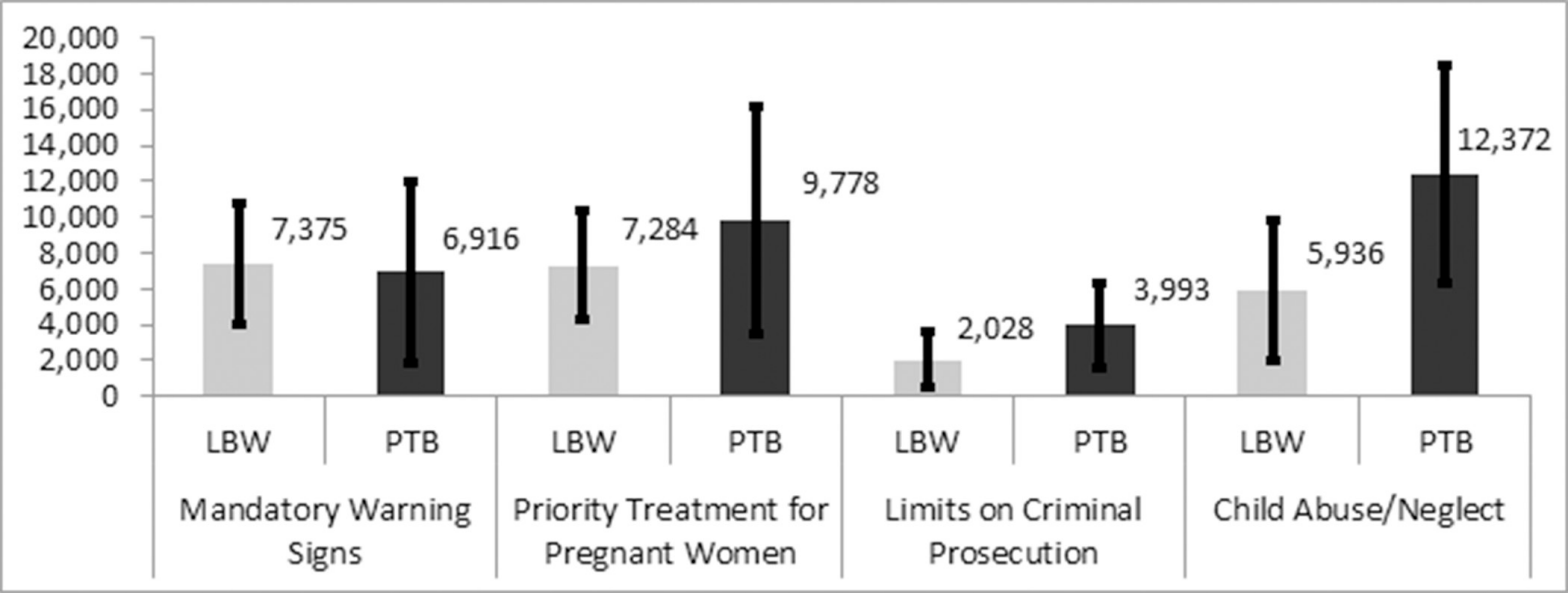
Numbers of excess low birthweight & preterm births due to state alcohol/pregnancy policies among babies born in 2015.

Table 1 displays the policies that led to significantly (*P* < 0.05) higher prevalence of LBW and PTB, and Fig 1 illustrates the number of excess LBW and PTB births associated with each. Given that mandatory warning signs and defining substance use during pregnancy as child abuse/neglect were the most widespread policies in place across the states in 2015, the numbers of babies affected appear largest for MWS, with an excess of 7,375 LBW, and for CACN with an excess of 12,372 PTB; these excess adverse outcomes are associated with an additional cost of $151,928,002 and $582,698,853 in the first year of life, respectively. The magnitude of effects were slightly smaller for PTPREG, which was associated with an excess of 7,284 LBW and 9,778 PTB, costing an additional $150,046,394 and $460,543,915 respectively. Finally, the effects of LCP were the smallest, with an excess of 2,028 LBW and 3,993 PTB babies costing an additional $41,779,191 and $188,072,680 in the first year of life.

**Table 1:** Excess numbers^a^ of low birthweight and preterm births with associated additional costs, 2015.

	Excess LBW (95% CI)	Cost^b^ (95% CI)	Excess PTB (95% CI)	Cost^c^ (95% CI)
**Mandatory Warning Signs**	7,375 (4,033, 10,717)	$151,928,002 ($83,080,108, $220,775,897)	6,916 (1,892, 11,940)	$325,749,619 ($89,093,136, $562,396,008)
**Priority Treatment for Pregnant Women**	7,284 (4,276, 10,292)	$150,046,394 ($88,081,231, $212,008,415)	9,778 (3,456, 16,100)	$460,543,915 ($162,767,241, $758,320,589)
**Limits on Criminal Prosecution**	2,028 (438, 3,618)	$41,779,191 ($9,025,653, $74,531,851)	3,993 (1,618, 6,369)	$188,072,680 ($76,188,248, $299,959,119)
**Child Abuse/Child Neglect**	5,936 (1,996, 9,876)	$122,285,003 ($41,115,560, $203,454,446)	12,372 (6,298, 18,544)	$582,698,853 ($291,945,927, $873,443,493)

^a^Among singleton live births in states with each individual policy in effect in 2015

^b^Assumes additional cost of low birthweight in first year of life is $20,600

^c^Assumes additional cost of premature birth in first year of life is $47,100

## Discussion

Multiple state alcohol/pregnancy policies–specifically MWS, PTPREG, LCP, and CACN–lead to thousands of babies born low birthweight or preterm each year. These increased rates of adverse birth outcomes cost hundreds of millions of dollars in health care and related costs annually. The actual prevalence and associated costs indicate that the harms related to alcohol/pregnancy policies are not only statistically significant, but also significant from a public health and public policy perspective. As most alcohol/pregnancy policies (with the exception of MWS) also apply to drugs [1], findings from this study for all policies other than MWS can be interpreted as applying to alcohol+drug/pregnancy policies rather than specific to alcohol/pregnancy policies. The MWS finding in particular, though, suggests that as states continue to legalize recreational cannabis, public health policy makers may want to exercise caution before expanding MWS to apply to cannabis.

Findings of adverse outcomes associated with MWS and CACN are plausible given previous literature [7, 10] showing that the fear of being reported to CPS and fear of having already irreversibly harmed ones baby are reasons women avoid prenatal care. However, explanations for findings related to both LCP and PTPREG are not as intuitive. One possible explanation comes from the historical context in which these particular policies emerged. Neither LCP nor PTPREG emerged as public health policies developed through a public health policy-making process, but rather emerged as advocacy arguments in response to the War on Drugs-related criminalization and punishment of pregnant women who used crack cocaine [19]. It is also worth noting that that limits on criminal prosecution (LCP) focuses primarily on limiting use of medical test results in criminal prosecutions related to alcohol/drug use during pregnancy–thus, states that adopt this policy could have more criminal prosecutions related to use during pregnancy. Priority treatment policies could be a marker of a state that does not have sufficient treatment slots either in general or for pregnant women, and thus the findings could be due to the lack of treatment availability for pregnant women, for women prior to becoming pregnant, or for women’s partners. Future research should examine these mechanisms. In the meantime, our findings strongly suggest that new policy approaches to alcohol/drug use during pregnancy are needed.

Strengths of this study include rigorously coded policy data and an outcome dataset that does not rely on self-report and that encompasses the entire population of singleton births for more than 40 years. Another strength is that analyses were able to incorporate state-specific time trends in addition to multiple individual and state-level controls and fixed effects for state and year. Adjusting for state-specific time trends helps address endogeneity and provides more confidence that the results we observe are due to the policies rather than the reverse. Limitations include that Vital Statistics data are not collected for research purposes, that they may not include key individual-level control variables, and that the measurement of some key variables (such as race and gestation) have changed over time. However, our adjustment for birth certificate version and inclusion of fixed effects for state and year alleviates concerns regarding changes in birth certificate data collection over time.

## Conclusions

Policymakers should consider the possibility of adverse public health impacts of alcohol+drug/pregnancy policies before expanding extant policies to new substances or adopting existing policies in new states.
